# Contaminations of Soil and Two *Capsicum annuum* Generations Irrigated by Reused Urban Wastewater Treated by Different Reed Beds

**DOI:** 10.3390/ijerph15081776

**Published:** 2018-08-18

**Authors:** Suhad A. A. A. N. Almuktar, Suhail N. Abed, Miklas Scholz

**Affiliations:** 1 Civil Engineering Research Group, School of Computing, Science and Engineering, The University of Salford, Salford M5 4WT, UK; suhad.suhad81@yahoo.com (S.A.A.A.N.A.); suhail.najem@gmail.com (S.N.A.); 2 Department of Architectural Engineering, Faculty of Engineering, The University of Basrah, Al Basrah 61001, Iraq; 3 Division of Water Resources Engineering, Department of Building and Environmental Technology, Faculty of Engineering, Lund University, P.O. Box 118, 22100 Lund, Sweden; 4 Department of Civil Engineering Science, School of Civil Engineering and the Built Environment, University of Johannesburg, Kingsway Campus, P.O. Box 524, Aukland Park 2006, Johannesburg, South Africa

**Keywords:** constructed wetland, cultivar, ecological engineering, irrigation water quality, sewage treatment, sustainable development, zinc

## Abstract

*Background*: In order to save potable water, this study aims to evaluate the contamination of soil and *Capsicum annuum* L. (chilli) watered with urban wastewater (sewage) pre-treated by various wetland systems. *Methods*: The appropriateness of wetland outflow for irrigation when applying reused wastewater with high contamination of minerals and pathogens was assessed. The impact of wastewaters pre-treated by various wetlands on soil and harvest was tested in terms of mineral and biological contamination risk. *Results*: The wetlands met the standards for irrigation water for most water quality variables. However, the thresholds for key water quality parameters were significantly (*p* < 0.05) exceeded. The highest values for total coliforms, ammonium-nitrogen, phosphorus and potassium were 157,072 CFU/100 mL, 8.5 mg/L, 5.0 mg/L, and 7.0 mg/L, respectively. The harvest was moderately polluted only by zinc according to vegetable quality standards (threshold of 50 mg/kg). Zinc concentrations for Filters 2, 4, 6, 7 and 8 were 35.8, 60.6, 65.1, 65.5 and 53.2 mg/kg, respectively. No bacterial contamination was detected. *Conclusions*: Treatment of domestic wastewater applying constructed wetlands and subsequent recycling of the treated wastewater for irrigation of crops is a good substitute to the traditional application of drinking water for irrigation purposes.

## 1. Introduction

### 1.1. Background

Solutions for worldwide challenges including water and food scarcity as well as pollution of water are needed. In developing nations, there is much interest in using reused treated wastewater for producing food. Benefits related to wastewater reuse comprise the supply of trace minerals and nutrients to plants, possibly resulting in yield increases and a reduction in the inorganic fertilizer need. Nevertheless, there are numerous problems linked to irrigation with reused urban wastewater. For example, increase in soil salinity with soil permeability reduction as well as toxic element and nutrient accumulation within watered soil and plants [1,2]. Furthermore, the elevated microbial level in the wastewater may result in both reduced harvest and crop quality as well as crops potentially contaminated with microbes [3].

Constructed wetlands of various designs including horizontal-flow and vertical-flow systems can treat various types of wastewater [4,5,6,7,8]. The evaluation of the effect of recycling of treated wastewater on crops to be consumed by humans requires attention. In Italy, recycling of treated domestic wastewater using constructed wetlands for subsequent crop irrigation has been applied according to Cirelli et al. [9]. The research shows that the treated wastewater was linked to high levels of *Escherichia coli* that considerably exceeded the local standard (50 colony forming units (CFU) per 100 mL).

The assessment of reused urban wastewater effects on irrigated crop quality and soil properties was carried out by Aiello et al. [10]. The results indicated that the use of wastewater led to increasing microbial pollution on the irrigated soil surface (*E. coli*: 3000 most probable number (MPN) per 100 mL; faecal *Streptococci* spp.: 1200 MPN per 100 mL). The disturbed soil layer was of low porosity as well as of decreasing hydraulic conductivity and water retention.

Moreover, the application of wastewater on soil for the assessment of potential contamination with heavy metals was undertaken by Chary et al. [11]. Findings indicated relatively high zinc, chromium and copper concentrations linked to the irrigated soil, which was available for growing plants.

There is a need for research assessing the effect of various wetland designs (including aggregate composition and planting) on treating domestic wastewater for subsequent crop irrigation reuse. The pre-treated wastewater has to be safe for consumption and not lead to soil contamination.

### 1.2. Motivation, Aim and Objectives

The main motivation for this experiment is to evaluate the contamination of soil and plants concerning pathogens and minerals that may affect the human health due to consumption. This article is built on work formerly published by Almuktar et al. [12,13], Almuktar and Scholz [7,14] and Almuktar et al. [8] concentrating on growing chillies with success to obtain their seeds for producing a new cultivar used to domestic wastewater. Almuktar et al. [8] dealt with the growth of daughter plants compared to their mothers. However, this new paper is concerned with soil and fruit contamination of chilli daughter plants and also fills a gap in scientific knowledge regarding the use of wetland systems to treat wastewater for subsequent agricultural food production.

Therefore, this project aims to evaluate the pollution of soil and two chilli generations (mothers and daughters) in terms of minerals and microbes due to irrigation with wastewater treated via numerous wetland systems. The objectives of this study were to: (a) assess the treated wastewater long-term suitability for irrigation of plants, (b) evaluate the impact of different wetland designs, operations and management practices on treated wastewater used for watering soil and fruits, and (c) compare yield quality subject to mineral and biological pollution risk between mother and daughter (i.e., first generation of a new cultivar) plants.

## 2. Materials and Methods

### 2.1. Set-Up and Experimental Operation

**Figure 1:**
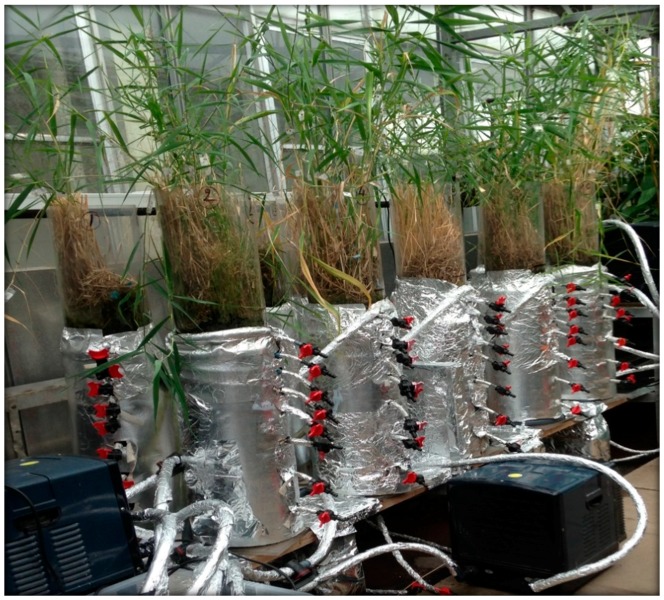
Experimental set-up of the experimental wetland system.

The experimental system (Figure 1) tested follows a completely randomised design and has been described by Almuktar and Scholz [6,7,12,13]. The experiment ran between 27 June 2011 and 25 September 2015 to study the influence of aggregate size and inflow loading rate as well as contact time (time the treated wastewater is in contact with the substrate) and resting time (time the substrate is not in contact with the treated wastewater; i.e., air fills the empty space between aggregates). The impact of aggregate diameter was tested when comparing Filters 1 and 2 (20 mm) with Filters 3 and 4 (10 mm), respectively. All contact times were 72 h, except for Filters 7 and 8 (36 h). All resting times were 48 h, except for Filter 8 (24 h).

The COD loading rate was another design parameter. Undiluted wastewater represented the high inflow loading rate. Wastewater diluted by 50% was used for the low loading rate. The controls received just chlorinated tap water. Therefore, the mean chemical oxygen demand (COD) inflow concentrations for Filters 1 to 4, 5, 6, 7 and 8 as well as the controls were 123.3, 244.7, 244.7, 123.3, 123.3 and 2.3 mg/L, respectively. The corresponding annually treated volumes of wastewater were 470, 470, 470, 624, 858 and 470 L/a in this order.

The constructed wetland Pyrex tubes had an inner diameter and height of 19.5 cm and 120 cm, correspondingly. Inert pea gravel was used to fill the tubes up to a depth of 60 cm. The wetlands were planted with *Phragmites australis* (Cav.) Trin. ex Steud., which is also known as common reed. The cleaned wastewater was harvested from the tubes via an outlet valve at the bottom as described, previously [4,6,8,12,13]. The preliminary treated urban wastewater came from the Davyhulme Sewage works located in Greater Manchester. Aqua Medic Titan chillers (Aquacadabra, Bexleyheath, UK) kept the wetland rhizosphere at around 12 °C [4].

The mother chilli plants were established from seeds purchased from B&Q plc (Chandlers Ford, UK) with the product code 03623879 on 14 September 2013 (experiment No. 1). The subsequent daughter chilli plant generation (experiment No. 2) was obtained from experiment No. 1.

The seeds were initially planted in shallow trays between 16 September and 10 October 2013, and then replanted (second planting between 11 October–7 November 2013) in nursery pots. The third (final) planting took place between 8 November 2013 and 25 September 2014. The more mature chillies were transferred to 10-litre pots (Hedgehogs Nursery, Glenrothes, UK) with the following dimensions: height, 22.0 cm; bottom diameter, 22.0 cm; and top diameter, 28.5 cm. B&Q plc provided the compost (product code 03717644). The planting depth was 17.5 cm. The soil was topped by 2.5 cm of bark (B&Q verve range, product code 5397007188110). The basic raw soil (original soil from supplier before use) properties were as follows: pH of 6.43, total nitrogen 999 mg/kg, total phosphor 368 mg/kg, potassium 2776 mg/kg, zinc 26.59 mg/kg and organic matter 89%. Finally, irrigation waters were obtained from Filters 1 to 8 as well as from Controls A and B. Concerning the experiment No. 2, the plants were grown in the compost using seeds randomly selected from the original mother plants obtained from experiment No. 1, which were irrigated with outflows from Filters 2, 4, 6, 7 and 8 only, because they had the best growth rate and yield quality [6,12,13,14].

The surface irrigation method was applied [1,15]. The soil moisture was constantly regulated using a graduated cylinder supplied by Fisher Scientific UK Ltd. (Loughborough, UK) according to Almuktar et al. [12,13] and Almuktar and Scholz [6,7]. The volume of treated waste water used for irrigating each plant was noted.

According to Almuktar et al. [12,13], a LUX meter ATP-DT-1300 (TIMSTAR, Winsford, UK) was used to regulate light. A thermometer-hygrometer-station supplied by wetterladen24.de (JM Handelspunkt, Geschwend, Germany) was applied to control temperature and humidity. The humidity was regulated using humidity meters (Challenge 3.0L Ultrasonic Humidifier; Argos, Central Milton Keynes, UK). Further details on the experimental methodology have been published by Almuktar et al. [8].

### 2.2. Quality Analysis of Water, Soil and Plants

All samples were tested according to APHA [16]. A Varian 720-ES Inductively Coupled Plasma —Optical Emission Spectrometer (ICP–OES; Agilent Technologies UK Ltd., Wokingham, UK) was applied to detect trace elements and nutrients in the samples [17]. A soil auger was used for soil sampling (to the depth of 20 cm) directly after the experiment. According to Chary et al. [11], the soil samples were assessed for mineral contents with the help of the Varian 720-ES Inductively Coupled Plasma—Optical Emission Spectrometer. Moreover, fruits were randomly selected for mineral content detection from each treatment after harvesting applying the Varian 720-ES Inductively Coupled Plasma—Optical Emission [18]. Faecal coliforms, total coliforms, faecal *Streptococcus* spp., *E. coli* and *Salmonella* spp. were detected for vegetables including flesh, skin and washing solution obtained from various spaces away from the irrigated soil as well as for the water and soil samples.

### 2.3. Analysis of Data

Microsoft Excel was used for simple analysis. The IBM‒SPSS Statistics Version 23.0 software (IBM, Armonk, NY, USA) calculated 5% significance levels. The normality was checked applying the Shapiro-Wilk test. The independent *t*-test was used for normally distributed data. In contrast, non-normally distributed data were tested applying the Mann-Whitney U-test. The one-way analysis of variance and the Kruskal-Wallis H-test were applied for data that were normally and non-normally distributed, respectively. The Spearman’s test helped with correlation studies.

## 3. Results and Discussion

### 3.1. Overview Regarding Irrigation Water Quality

#### 3.1.1. Oxygen Demand Parameters

Table 1 indicates highest COD values for Filter 6 followed by Filter 8, and the lowest concentrations for Filter 7. Almuktar et al. [8] indicated no significant differences (*p* > 0.05) in COD values for Filters 2 and 4 compared to Filters 4 and 7 in this order. It follows that aggregate size and contact time did not really matter statistically with respect to COD removal. The COD values for Filters 7 and 4 were significantly (*p* < 0.05) different compared to the concentrations of Filters 8 and 6, correspondingly, explaining the impact of resting time and inflow loading rate.

The five-day biochemical oxygen demand (BOD) was the highest for Filter 6 followed by Filter 2. In comparison, Filter 7 had the lowest BOD. The BOD concentrations of Filters 2 and 4 were higher with respect to those of Filters 4 and 7, correspondingly. This showed the influence of aggregate size and contact time. Filter 7 had BOD values lower than those concerning Filter 8. This explains the impact of resting time. The BOD for Filter 6 was significantly (*p* < 0.05) higher compared to Filter 4 [8] supporting findings by Sani et al. [4], stating that high-load filters are commonly under-performing. The dissolved oxygen was highest for Filter 4 followed by Filters 8 and 7. In comparison, the Filters 2 and Filter 6 were deprived of oxygen. However, Almuktar et al. [8] did not reveal any significant differences between filters.

#### 3.1.2. Irrigation Water Nutrients and Trace Elements

The highest ammonium-nitrogen values were recorded for Filter 6 followed by Filters 2 and 7 (Table 1). In comparison, the lowest values were linked to Filter 8 followed by Filter 4. Filter 2 concentrations were significantly (*p* < 0.05) higher than those for Filter 4 (Table 1 and [8]) showing the influence of aggregate diameter and nitrogen transformation processes. Ammonium-nitrogen values for Filter 4 were lower than for Filter 7, while the latter outflow water had ammonium-nitrogen concentrations significantly (*p* < 0.05) higher compared to those for Filter 8. This indicates a great impact of both contact and resting times on ammonium. Elevated COD inflow water led to significant (*p* < 0.05) differences between the ammonium-nitrogen concentrations between Filters 4 and 6 [8], indicating that high-load filters are underperforming [4]. High ammonium-nitrogen concentrations above standard thresholds were noted for Filter 6 (high inflow loading rate), impacting negatively on plant fruit, leaf and growth [8,19].

High nitrate-nitrogen concentrations were linked to Filter 6 and low ones to Filter 4 (Table 1). Almuktar et al. [8] indicated that nitrate-nitrogen values were significantly (*p* < 0.05) greater for Filter 2 in comparison to Filter 4. This can be explained by different aggregate diameters. Significant differences (*p* < 0.05) in nitrate-nitrogen outflows of Filters 4 and 7 were recorded. This can be explained by the influence of contact time. Filters with a higher inflow loading rate had nitrate-nitrogen concentrations that were significantly (*p* < 0.05) greater compared to those subjected to diluted inflow (i.e., Filter 4 compared to Filter 6) as shown previously by Sani et al. [4].

Nitrate-nitrogen for all filter outflows was below the threshold of 30 mg/ L [1,20]. The total yield increases as the nitrate-nitrogen to ammonium-nitrogen ratio elevates. This can be explained by a reduction in fruit physiological disorders decreasing fruit weights [19].

The highest orthophosphate-phosphorus concentrations were noted for Filter 6 followed by Filter 7. In contrast, the lowest corresponding concentrations were obtained for Filter 4 (Table 1). Filter 2 had orthophosphate-phosphorus concentrations higher than those for Filter 4 (Table 1). This can be explained by a lower removal rate due to less active biomass within the spaces between aggregates. Filter 4 had lower orthophosphate-phosphorus concentrations than Filter 7. However, Filter 7 had concentrations higher than the ones for Filter 8 due to the influences of different wetland contact and resting times on orthophosphate-phosphorus concentrations.

Irrigation water linked to Filter 6 had orthophosphate-phosphorus concentrations that were significantly (*p* < 0.05) higher compared to those of Filter 4 [8]. This can be explained by the impact of the inflow loading rate on orthophosphate-phosphorus, which is difficult to remove [5,19,21]. Compared to the standard threshold of 2 mg/ L, all recycled outflow waters had orthophosphate-phosphorus values exceeding the threshold [1,20]. Findings indicate that phosphorus deficiency limits crop yields and enhances the uptake of manganese [22,23].

An ICP—OES analysis for the irrigation water based on a sample number of 15 has been performed, previously [8]. Elements such as arsenic, barium, bismuth, cadmium, cobalt, chromium, copper, nickel, lead, strontium and titanium were below the detection limits. High iron for Filter 7 followed by Filter 6 was noted. The lowest concentrations were recorded for Filter 4. In comparison, Filter 2 had iron values higher than those for Filter 4. This can be explained by the impact of wetland aggregate diameter on the removal of iron. Filters 4 and 7 had iron concentrations significantly (*p* < 0.05) different from those of Filters 7 and 8, respectively [8], indicating the impacts of contact and resting times on iron removal. Filter 6 had iron values that were significantly (*p* < 0.05) higher than those of Filter 4 [8]. However, all concentrations were below the threshold of 5.0 mg/ L [1,20].

Iron is removed by oxidative processes and iron hydroxide creation [24,25]. However, biotic processes involving bacteria in iron oxidation can also be important [25]. Oxygen and pH are essential variables for iron removal processes influenced by photosynthesis.

Magnesium concentrations were highest for Filter 7 followed by Filter 6. Low numbers were observed for Filter 2. Filter 4 had manganese concentrations, which were significantly (*p* < 0.05) lower compared to Filter 7. Moreover, Filter 7 values were significantly (*p* < 0.05) higher than those for Filter 8, highlighting the impact of both contact and resting times on manganese reductions. An elevated loading rate significantly (*p* < 0.05) impacted on the outflow water iron concentration. This can be seen when comparing Filter 4 to 6 (Supplementary Materials Table S1). Manganese concentrations were usually below the corresponding threshold of 0.2 mg/ L [1,20].

Manganese reductions are due to oxygenic photosynthesis particularly during summer. However, iron should be removed first to support manganese elimination [25]. High manganese values are linked to low plant growth [26]. Manganese phyto-toxicity reduces biomass and photosynthesis [27].

Zinc within irrigation waters was highest for Filter 6 followed by Filter 2. The lowest concentrations were linked to Filter 4. However, Almuktar et al. [8] indicated that there were no significant differences in zinc concentrations associated with different aggregate diameters, contact times, resting times and inflow loading rates [6,7], complementing similar results published elsewhere [28]. All zinc outflow concentrations were below the standard of 2.0 mg/ L [1,20].

The highest boron concentrations for Filter 6 were followed by Filter 2. In comparison, the lowest boron concentrations were noted for Filter 7. Nevertheless, Almuktar et al. [8] showed no significant (*p* > 0.05) differences for boron concerning all wetlands [6,7]. Moreover, all boron concentrations were lower than the standard of 0.75 mg/L [1,15].

Filters 6 and 4 had the highest and lowest potassium values, respectively. Filter 2 (large aggregates) had potassium concentrations greater than those for Filter 4 (small aggregates). Filter 7 had potassium values greater than those for Filters 4 and 8 due to differences in contact and resting time in this order. This can be explained by the influence of aggregate diameter, contact time and resting time on potassium removal [6,7]. Wetlands with undiluted wastewater had potassium concentrations that were significantly (*p* < 0.05) greater than those of diluted inflow (e.g., comparison between Filters 6 and 4). This observation agrees with the literature [4] highlighting that filters of elevated inflow loading rate are commonly overloaded. All outflows exceeded the potassium threshold of 2.0 mg/L [1,15].

Relatively high potassium concentrations protect plant stems from damage during cold nights [29]. Furthermore, frost reduces the harvest and increases leaf damage, which can be addressed by increasing the potassium concentrations in the irrigation waters. Furthermore, elevated potassium lowers seedling death rates during cold periods [29].

Sodium values were highest for Filter 6 followed by Filter 7, and lowest for Filter 8. Drain waters from Filters 2, 4, 7 and 8 had rather similar concentrations. Almuktar et al. [8] indicated that there were significantly (*p* < 0.05) lower values for Filter 4 compared to Filter 6, because of differences in terms of the inflow loading rate [4]. All wetland sodium concentrations were below the standard of 920 mg/ L [1,20].

Calcium values were highest for Filter 6 followed by Filter 4. In contrast, the lowest concentrations were recorded for Filter 2. Filter 2 (large gravel) had calcium concentrations lower than the values for Filter 4 (small gravel). Moreover, Filter 4 had calcium values higher than those for Filter 7 due to different contact times. Filter 4 was fed with diluted wastewater, and had calcium values lower than those for Filter 6 (high inflow load). All irrigation waters had calcium values lower than the threshold of 400 mg/ L [1,20].

The highest magnesium concentrations were associated with Filter 6 and the lowest ones with Filter 8. Almuktar et al. [8] showed that Filter 6 had magnesium values, which were significantly (*p* < 0.05) higher compared to those obtained from Filter 4. All irrigation waters had values that were below the standard of 60 mg/ L for magnesium [1,20]. The removal can be explained by processes such as sorption by sediment, adsorption, complexation, cation and anion exchange, oxidation and reduction, chemical precipitation, co- precipitation as insoluble salts and phyto-accumulation as well as microbial and plant uptake [30,31,32,33].

#### 3.1.3. Particles, pH and Salinity

The highest suspended solids and turbidity values were noted for Filter 2 followed by Filter 6 (Table 1). In contrast, lowest numbers were noted for Filter 8. Irrigation waters obtained from Filter 2 had suspended solids and turbidity values that were significantly (*p* < 0.05) greater than those for Filter 4 [8]. This is due to the impact of wetland aggregate size on processes such as filtration [4]. Filter 7 had suspended solids and turbidity values greater than those of Filter 8. This can be explained by differences in resting time. The loading rate significantly (*p* < 0.05) impacted on the particle content (e.g., Filter 6 compared to Filter 4 [8]).

High suspended solids and turbidity increase soil hydrophobicity [6,7,12,13,33]. Previous studies [34,35,36,37] indicated that solids can be removed via sedimentation, settling, adsorption and biological degradation processes. Furthermore, in surface flow systems, suspended solids may be reduced via flocculation, sedimentation and filtration processes [38]. Suspended solids also interact with heavy metals, nutrients, pathogens and organic matter, which lead to combined removal processes [39]. In sub-surface vertical-flow constructed wetlands, substrate, hydraulic load and microorganisms determine solids removal [40].

Recorded pH values were normal [1,20]. Filter 6 had the highest pH values followed by Filter 7. In contrast, the lowest numbers were recorded for Filter 2 (Table 1). Almuktar et al. [8] showed that there were significant (*p* < 0.05) differences in pH recordings of outflow waters subject to contact time, resting time and the inflow loading rate.

The pH commonly impacts on nitrogen and organic matter removal. The reduction of alkalinity during nitrification will lead to a drop in pH, which affects the denitrification rate [34]. The optimum pH for denitrification ranges from 6 to 8. The highest rate has commonly been observed at a pH value between 7.0 and 7.5 [41].

Electrical conductivity correlates well with salinity [15]. The highest conductivity was noted for Filter 6 followed by Filter 8 (Table 1). In contrast, the lowest numbers were recorded for Filter 2. Filter 6 had salinity values that were significantly (*p* < 0.05) greater than those for Filter 4. This is due to overloading [4]. Nevertheless, all recorded salinity values were below the standard of 3000 µS/cm [1,20]. An elevated electrical conductivity results in saline soil, which is negative for plant growth, soil structure and soil permeability [42].

#### 3.1.4. Microbial Indicators within the Irrigation Water

The key indicators of microbial contamination detected in the irrigation water have been discussed, previously [8]. The highest total coliform recordings are associated with Filter 6 followed by Filter 2. The lowest ones were noted for Filter 8. Filter 4 had total coliform numbers that were significantly (*p* < 0.05) different from the ones for Filters 6 and 7 [8]. Differences in filter loading rate and contact time are responsible for this observation. Filter 7 had total coliforms higher than those for Filter 8, explaining the impact of resting time on the wetland system. All irrigation waters exceeded the standard of 1000 CFU per 100 mL [43].

*Escherichia coli* contamination followed this order: Filter 6 > Filter 2 > Filter 8 [8]. Irrigation water harvested from Filter 2 (large aggregates) had *E. coli* values (71 CFU per 100 mL) greater than those (0 CFU per 100 mL) for Filter 4 (small aggregates). Filter 6 had *E. coli* numbers (5572 CFU per 100 mL) greater than those (0 CFU per100 mL) for Filter 4. Furthermore, Filter 8 (long resting period) had *E. coli* numbers (59 CFU per 100 mL) higher than those for Filter 7 (0 CFU per 100 mL) of short resting periods. Filters 4 and 7 were not contaminated by *E. coli*, which was not expected [9].

*Salmonella* spp. counts were high for Filter 6 followed by Filter 2 [8]. The lowest numbers were recorded for Filter 7. Filter 4 counts were significantly (*p* < 0.05) higher compared to Filter 7 [8] indicating the influence of contact and resting times on microbial contamination. Furthermore, Filter 6 had significantly (*p* < 0.05) higher counts than Filter 4 due to different loading rates [8], which confirms previous findings [4,14].

Wetlands with large aggregate diameters had higher *Salmonella* spp. counts than those with small aggregates. This is due to large aggregate diameters permitting more organisms to colonies the space between stones [6,7,14].

### 3.2. Analysis of Soil Quality

#### 3.2.1. pH and Redox Potential Comparison

**Figure 2:**
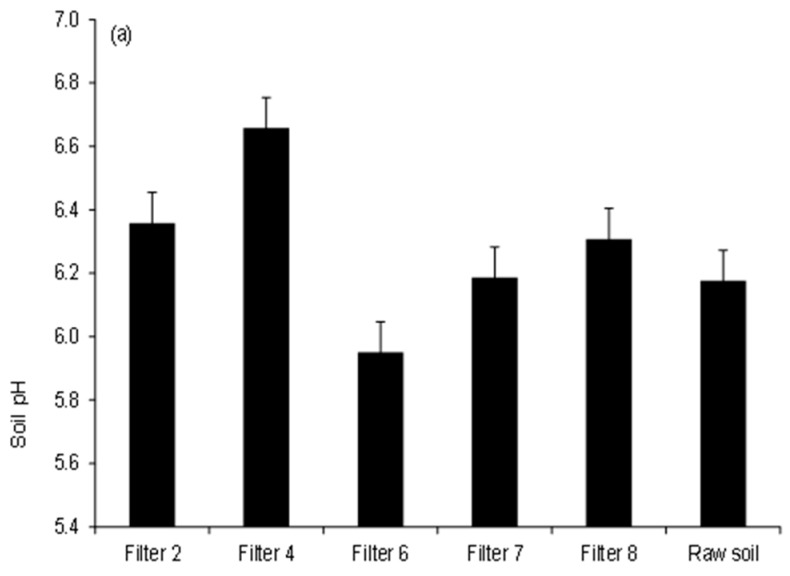

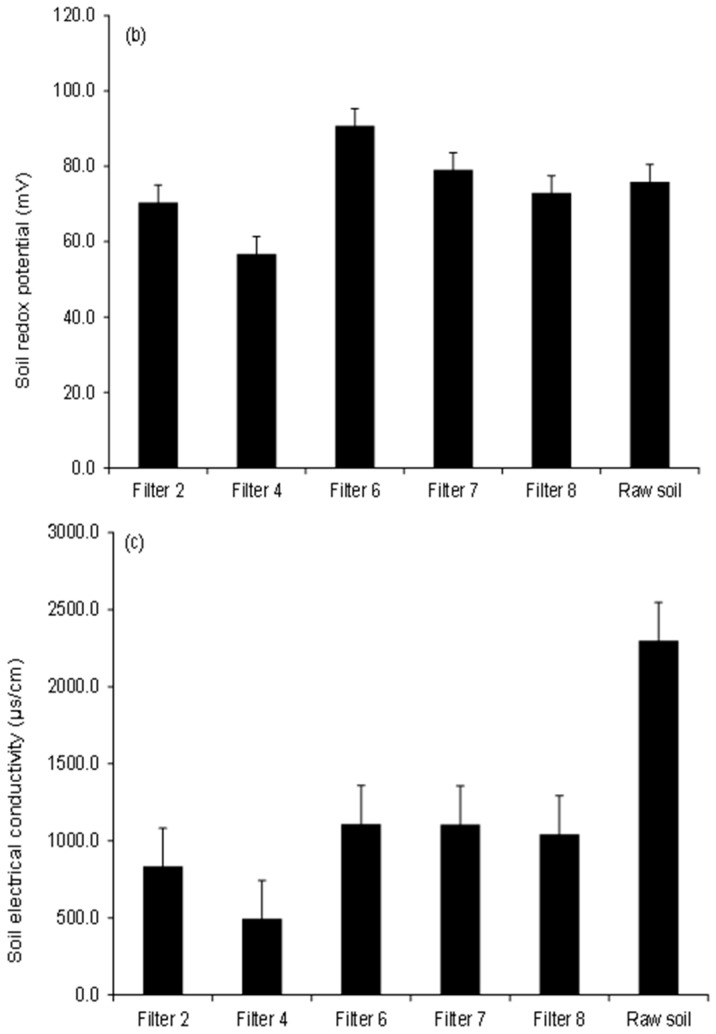
Soil qualities subject to irrigation water: (**a**) pH; (**b**) redox potential, and (**c**) electrical conductivity.

Figure 2 illustrates qualities for soils irrigated with wastewater treated by wetland filters. Ten soil samples were tested. All soil pH values showed acidic conditions, impacting on plant trace element uptake [1,23]. Supplementary Materials Table S2 indicates that compared with raw soil (pH = 6.2), irrigation with pre-treated wastewater did not cause significant (*p* < 0.05) changes with the exception of soil irrigated with Filter 4 outflow, which showed the highest pH values of 6.7 tailed by those of Filter 2 (pH = 6.4), while minimum values were reported for soils watered with Filter 6 effluent showing a pH value of 6.0. 

Furthermore, irrigation with Filter 4 drain water showed pH values that were significantly (*p* < 0.05) greater compared to those for Filters 2, 6 and 7, while no significant differences (*p* > 0.05) were observed, if pH values of soil watered with Filters 7 and 8 drain waters are compared with each other. However, all soil pH entries were within the optimal range of from 6.0 to 7.5 for growth of many plants and soil microbes [1,23].

Regarding soil redox potential values, soil can be classified as moderately reduced soil (+100 to +400 mV), reduced soil (−100 to +100 mV) and highly reduced soil (−100 to −300 mV), according to Husson [44]. Figure 2 indicates that all soil could be interpreted as reduced. The highest redox potentials were noted for soils watered with Filter 6 effluent followed by those linked to Filter 7 showing values of 90.8 mV and 79.1 mV, respectively, while the lowest values were associated with soils irrigated with water harvested from Filter 4 (redox potential value of 56.8 mV). Results showed that irrigation with treated wastewaters did not change soil redox potential values significantly (*p* > 0.05) compared with the raw soil (Supplementary Materials Table S2).

Moreover, soils watered with Filter 4 effluent showed redox potential values that were significantly different (*p* < 0.05) from those of Filters 2, 6 and 7, while no dissimilarities (*p* > 0.05) were calculated when making a comparison between redox potential values of soil watered with Filter 7 with those corresponding ones linked to Filter 8. High redox potentials can impact on the health of plants [43]. Furthermore, correlation analysis results showed that both pH and redox potential values were significantly negatively (*R* = −0.986, *p* < 0.001) correlated with each other, as reported by FAO [23] and Essington [45].

#### 3.2.2. Soil Salinity Comparison

Figure 2 shows that irrigation with pre-treated wastewater did not result in an increase of the soil salinity, if compared to the raw soil. However, among the irrigated soils, application of outflow water from Filter 6 (high inflow loading) resulted in the highest electrical conductivity value of 1108 µS/cm, while soil linked to Filter 4 showed the lowest value of 493.8 µS/ cm. Moreover, salinity values for soil irrigated with Filters 2 and 7 effluents were greater than those of Filters 4 and 8, respectively. Irrigation with Filters 6 and 7 effluents resulted in soil salinity values, which were significantly (*p* < 0.05) greater than those for Filter 4 (Supplementary Materials Table S2), highlighting the impact of wetland system design in terms of both contact time and inflow loading rate on the salinity of the outflow water (Table 1). Furthermore, correlation analysis results showed that soil electrical conductivity values were significantly negatively correlated with soil pH values (*R* = −0.899, *p* = 0.015). For example, in acidic conditions of low pH value, the dissolution of elements such as calcium, potassium, sodium and magnesium will increase resulting in elevated soil salinity. However, excessive soil salinity could result in nutrient imbalances [1,23].

#### 3.2.3. Soil Microbes

**Figure 3:**
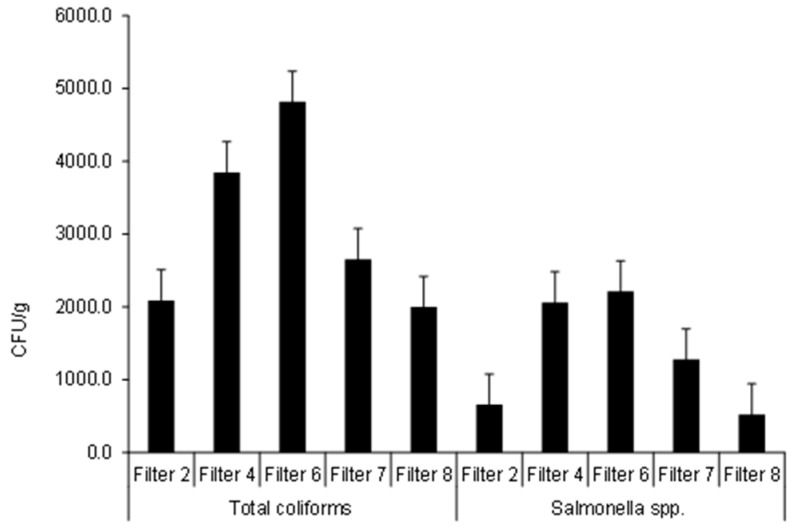
Soil microbial content.

Figure 3 shows that total coliforms were detected with the highest values in the soil watered with Filter 6 effluent (4818 CFU/g) followed by those linked to Filter 4 showing a value of 3846 CFU/g. *Streptococci* spp. and *E. coli* were not detected in the tested soils. Ten soil samples were analyzed. The lowest values were associated with soils irrigated with Filter 8 (2000 CFU/g). Supplementary Materials Table S2 highlights that soil watered with Filter 4 effluent had total coliform colonies significantly (*p* < 0.05) higher than those soils irrigated with Filters 2 and 7 effluents, showing the impact of stone diameter and contact time of the filter on outflow water total coliforms and subsequently on the irrigated soils. Irrigation with outflow water from an undiluted inflow wetland filter significantly (*p* < 0.05) increased soil total coliform levels compared with those of diluted inflow wastewater as shown when comparing soils irrigated with Filter 6 with those irrigated with Filter 4 effluent (Supplementary Materials Table S2).

Similar to the total coliform development, Figure 3 shows that the highest *Salmonella* colonies were detected in soil watered with Filter 6 followed by those irrigated with the effluent of Filter 4, showing values of 2214 CFU/g and 2062 CFU/g, respectively. The lowest values were noted for soils irrigated with Filer 8 outflow water (524 CFU/g). Statistically, soil watered with water drained from Filter 2 of large aggregate size had *Salmonella* counts significantly (*p* < 0.05) lower than those linked to Filter 4 of small aggregate diameter, in spite of their high abundancy in the outflow waters of the former [8]. Significant differences (*p* < 0.05) in irrigated soil *Salmonella* counts were also observed when comparing soils associated with Filters 4 and 7 with those of Filters 7 and 8, respectively, due to differences in contact time and resting time design variables for the corresponding filters in the wetland system (see above). Correlation analysis findings indicated that both total coliforms and *Salmonellae* were significantly positively correlated with each other (*R* = 0.954 and *p* value of less than 0.001), confirming results reported by Almuktar and Scholz [14]. The survival time of soil bacteria in crops and water is less than 70, 30 and 60 days in this order [46].

#### 3.2.4. Soil Aluminum

**Figure 4:**
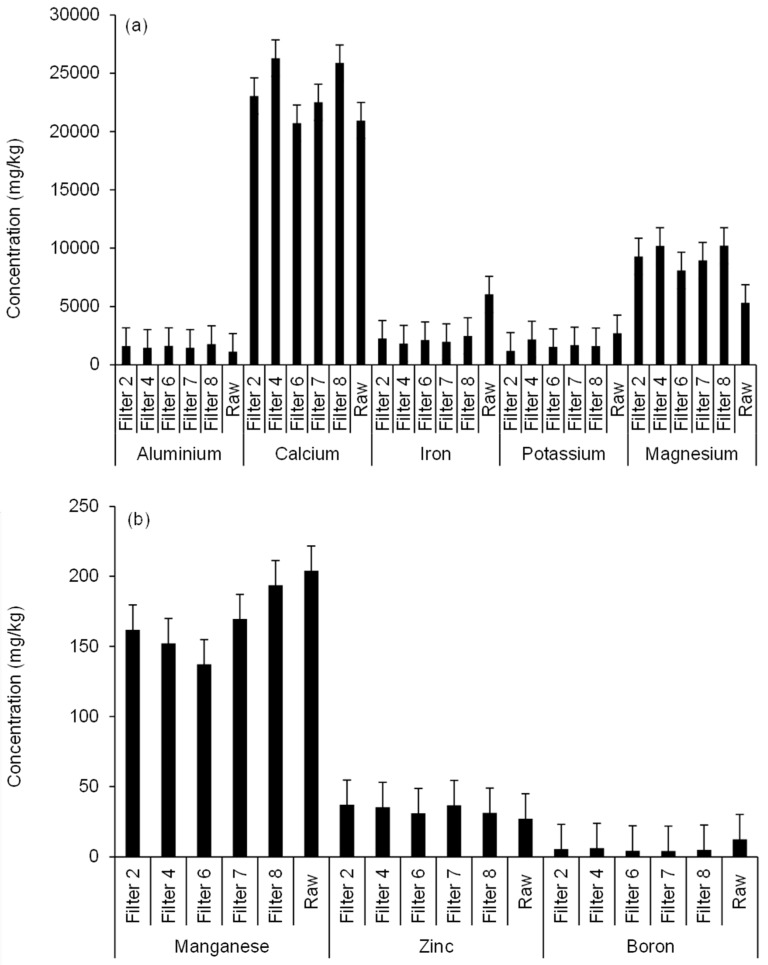
Inductively coupled plasma optical emission spectrometer analysis for elements in the irrigated soil: (**a**) aluminum, calcium, iron, potassium and magnesium; and (**b**) manganese, zinc and boron.

The solubility of aluminum is influenced by pH, organic compounds and clay content of the soil. When the soil pH decreases, exchangeable aluminum will instantly increase. The findings showed that watering with wetland treatment system drain water significantly (*p* < 0.05) increased aluminum levels, if compared to the raw soil (Figure 4 and Supplementary Materials Table S2) due to its rather low biological availability [47]. Concerning Figure 4, the sample number was 24. Note that other elements such as arsenic, barium, bismuth, cadmium, chromium, cobalt, copper, nickel, lead, strontium and titanium were below the detection limit. Results showed that the maximum aluminum concentrations were detected in soil watered with Filter 8 (followed by Filter 6) effluent, while minimum values were recorded for soils irrigated from waters obtained from Filters 4 and 7. Filter 4 outflow water irrigation resulted in mean aluminum concentrations lower than those for soils linked to Filters 2 and 6, showing the impact of wetland filter aggregate diameter and inflow loading rate on outflow water aluminum concentrations and subsequently on its levels in the watered soil. Furthermore, soils watered with Filter 7 drain water had aluminum values, which were significantly (*p* < 0.05) lower than those for Filter 8 (Figure 4 and Supplementary Materials Table S2), probably due to the difference in applied volumes of irrigation water [8]. Correlation analysis results (Supplementary Materials Table S3) show that aluminum concentrations in the irrigated soil were significantly (*p* < 0.001) positively correlated with calcium, iron, potassium, magnesium, manganese and zinc values, while adversely correlated with boron levels in the soil, as informed by Essington [45].

#### 3.2.5. Soil Zinc

Figure 4 indicates that maximum zinc values were recorded for the soil watered with water drained from Filters 2 and 7. In comparison, minimum concentrations were noted for those soils watered with Filter 6 effluent. Compared to the raw soil, no significant (*p* > 0.05) increase in the irrigated soil zinc levels were noted (Supplementary Materials Table S2). Irrigation with outflow water from Filter 2 (large stones) caused higher soil zinc values compared to those soils watered with from Filter 4 (small stones) as can be seen in Figure 4. Moreover, soils watered with effluents from Filters 4 and 7 had zinc levels, which were dissimilar from those linked to Filters 7 and 8, correspondingly, because of differences in their contact time and resting time variables of the systems (see above). However, no significant differences (*p* < 0.05) in mean soil zinc concentrations among soils irrigated with differently treated wastewaters were noted (Supplementary Materials Table S2). These results indicate the effect of wetland system design variables on the treated water zinc concentrations [8] leading to differences in the distribution of this element in the irrigated soils. Zinc concentrations in the irrigated soil were significantly (*p* < 0.001) positively correlated with calcium, iron, potassium, magnesium, manganese and aluminum values (Supplementary Materials Table S3), while negatively correlated with boron levels in the soil [45]. Nonetheless, concentrations of zinc in watered soil were below the threshold of 300 mg/kg [48].

#### 3.2.6. Soil Boron

There are numerous factors that may affect boron soil availability like soil texture, nature of the soil minerals, source of the irrigation water, organic matter content, soil pH, environmental conditions (mainly high light intensity and dry weather) and the relationships of boron with other elements [23]. Figure 4 illustrates that the maximum boron concentrations were detected in soil watered with Filter 4 followed by Filter 2 effluent. Minimum concentrations were reported for soil watered with effluent from Filter 7. In comparison with raw soil, watering with treated wastewaters did not elevate the soil boron levels (Figure 4). Findings indicate no significant (*p* < 0.05) differences in irrigated soil boron values (Supplementary Materials Table S2). However, soil watered with effluent from Filter 4 had higher boron concentrations compared to those soils watered with Filters 2, 6 and 7 outflows. Also, soils watered with Filter 7 drain water had boron levels lower than those watered with effluent from Filter 8. This indicates the effect of wetland system design variables on treated water boron concentrations [8], which caused differences in the element distribution, when applied to soils. Correlation analysis (Supplementary Materials Table S3) showed that boron concentrations in the soil correlated negatively with other elements in the soil, as discussed by Essington [45].

#### 3.2.7. Other Elements

Refer to Almuktar et al. [8] concerning total element masses in general and calcium, iron, potassium, magnesium and manganese contaminants in particular, which are usually not a problem in soil.

### 3.3. Chilli (First Generation) Microbial and Mineral Contents

#### 3.3.1. Overview

**Figure 5:**
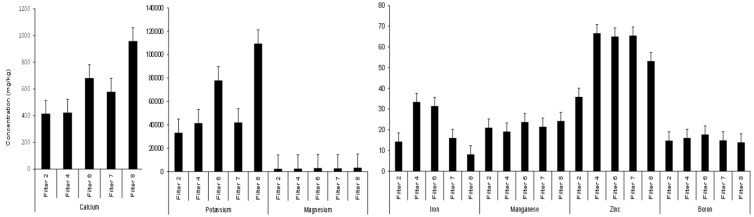
Elemental analysis within harvested chili daughter generations.

Harvested chillies (both skin and flesh) as well as the corresponding washing solution were free from microbial contamination. This is due to the relative long distance between the potentially contaminated soil and the harvested fruits [9]. This confirms previous results [14]. Regarding chilli mineral contamination, Figure 5 and Supplementary Materials Table S4 show the detected element concentrations in chillies obtained from first generation plants and the associated statistical analysis results, respectively. The bioavailability of each element was indicated by calculating the concentration factor (CF) value. This factor, as a percentage, is defined as the relationship between element concentration in the plant organ and its concentration in the soil [49] as shown in Equation (1):

CF = 100* (C fruit C soil^−1^) (1)

where CF is concentration factor (%), C fruit is the element concentration in the fruit (mg/kg), and C soil is the element concentration in the soil (mg/kg)

Findings showed that the CF values in the chillies followed the order potassium > boron > zinc > magnesium > manganese > calcium > iron, with percentage values of 3819, 314, 169, 30, 14, 3 and 1, respectively. More details on fruit element content are shown below.

#### 3.3.2. Aluminum

The solubility of aluminum depends on the soil pH and its content in terms of clay and organic material. Figure 5 shows that aluminum was not observed in any of the harvested fruits from the first generation plants watered with different water types [6]. According to Husson [44], aluminum concentrations can be increased at low pH. Findings indicated that an abundancy of aluminum was detected in the irrigated soils (Figure 4). Despite of this, the transfer of aluminum to the tissue of fruits was limited. This is possibly due to the great abundance of calcium (see below) in the watered soil, which limited aluminum transportation to the plants [23]. Regarding human health, Stahl et al. [47] showed that there is no risk linked to the presence of too much aluminum due to its low bioavailability [50].

#### 3.3.3. Calcium

**Figure 6:**
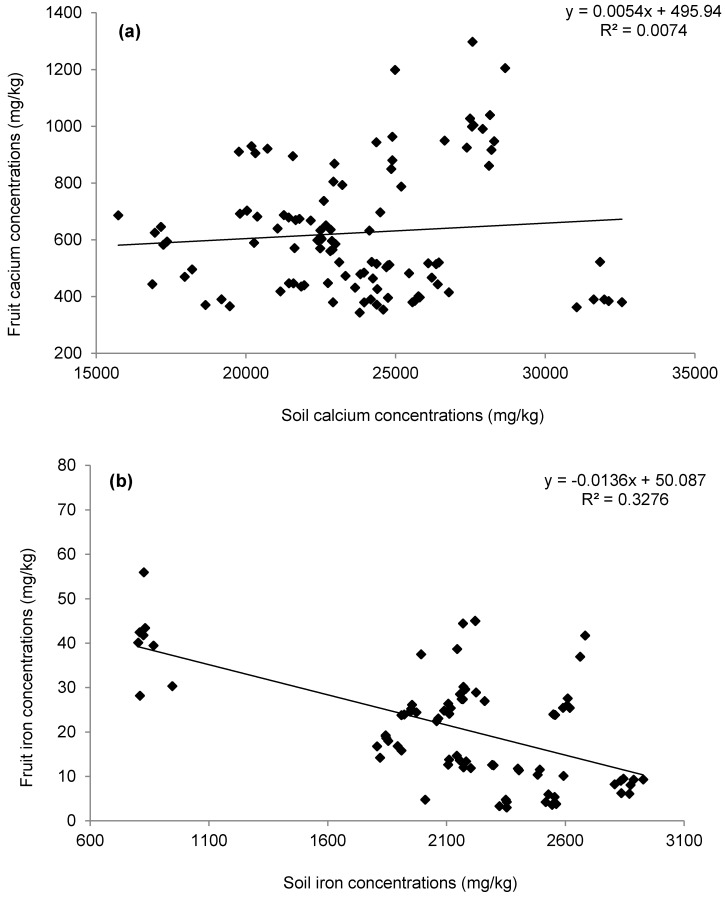

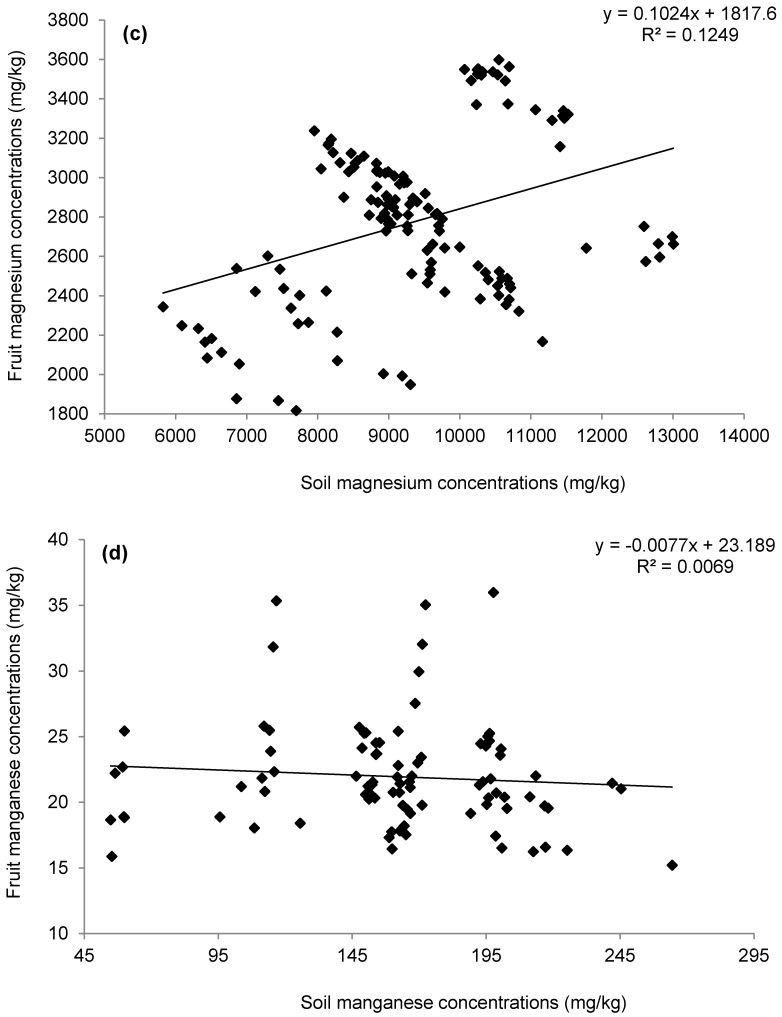

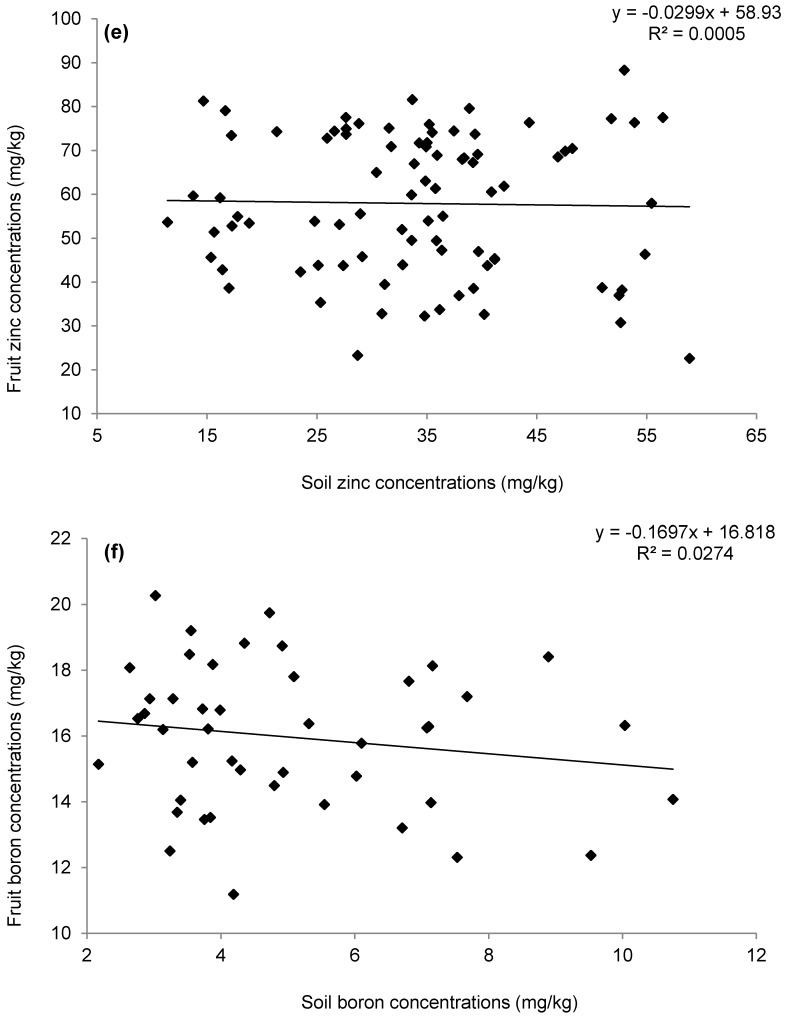
Influence of soil on fruits concerning the elements (**a**) calcium; (**b**) iron, (**c**) magnesium, (**d**) manganese, (**e**) zinc, and (**f**) boron.

Figure 5 indicates that maximum calcium values were detected in fruits obtained from chillies watered with Filter 8 effluent, trailed by those irrigated with Filter 6 harvested water. On the other hand, minimum calcium concentrations were noted for the fruits of chillies watered with Filter 2 effluent. However, calcium levels in all tested chilli fruits (except for those of Filters 2 and 4) were above 45 mg per 100 g [51]. Supplementary Materials Table S4 shows that plants watered with Filter 2 (large stones) drain water produced chillies of calcium concentrations, which were similar to those associated with Filter 4 (small stones). Moreover, irrigation with the effluent of the high-load Filter 6 resulted in fruit calcium concentrations, which were significantly (*p* < 0.05) greater than those associated with Filter 4 (subjected to diluted wastewater) as shown in the Supplementary Materials Table S4. Furthermore, chillies linked to Filter 7 had calcium concentrations that were significantly (*p* < 0.05) different from those linked to Filters 4 and 8. No significant (*p* > 0.05) correlation was calculated for calcium values between soil and fruits (Figure 6a).

**Figure 7:**
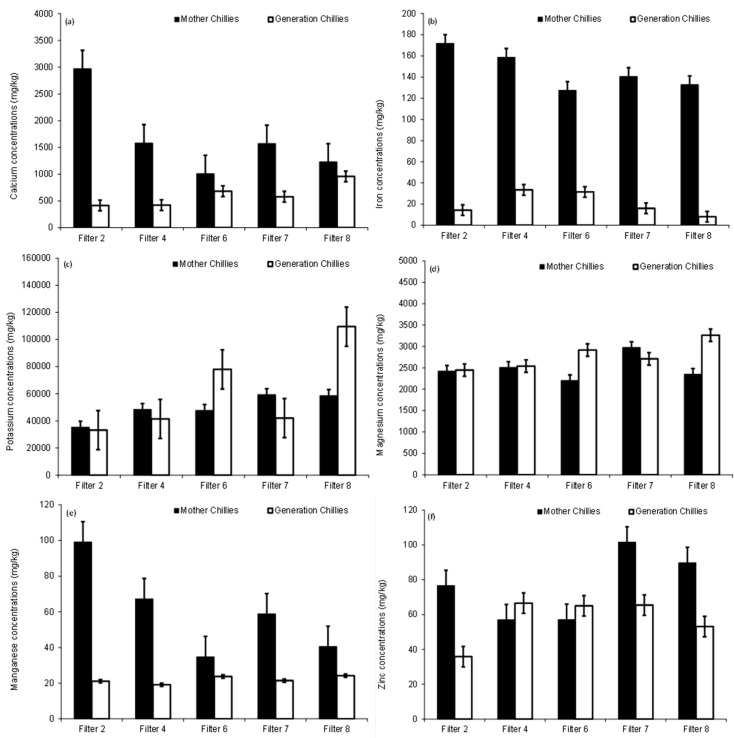
Chilli generations: (**a**) calcium, (**b**) iron, (**c**) potassium, (**d**) magnesium, (**e**) manganese, and (**f**) zinc.

Moreover, the calcium CF in the tested fruits had this order: Filter 8 > Filter 6 > Filter 7 > Filter 2 > Filter 4 with percentages of 3.7%, 3.3%, 2.6%, 1.8% and 1.6%, respectively. Compared with their mothers, the first chilli generation fruits had calcium concentrations that were significantly (*p* < 0.05) less (Supplementary Materials Table S4 and Figure 7a), explaining the impact of higher irrigation water amounts applied to mother plants than those used for the first generation plants [8]. However, calcium in the soil competes with other major cations such as sodium, potassium, magnesium, ammonium, iron, and aluminum for the uptake by crops [23]. High potassium loads lower the calcium uptake in plants [52].

As the soil pH goes down, mainly iron (Fe^2+^) and aluminum (Al^3+^) become soluble and react with calcium to form insoluble compounds [52]. High calcium levels may inhibit boron uptake. Calcium is essential for many plant functions [23]. From a human health point of view, calcium plays a main role in bone structure, metabolism processes, muscle and nerve function control as well as balancing of blood streams as reported by Zhu and Prince [53].

#### 3.3.4. Iron

Figure 5 illustrates that maximum iron values were detected in fruits from chillies receiving effluent from Filter 4, followed by those fruits irrigates with Filter 6 effluent. Minimum iron numbers were noted in fruits of chilies watered with Filter 8 outflow. The detected iron levels in fruits obtained from all treatments were considerably lower than 6.04 mg per 100 g of dried chillies as reported by Ciju [51]. Statistical results (Supplementary Materials Table S4) indicate that the effect of the wetland aggregate diameter was significant (*p* < 0.05), if compared to the mean iron values in fruits harvested from chillies irrigated with outflow from Filters 2 and 4.

Fruits taken from chillies watered with effluent from Filters 4 and 7 had mean iron values that were significantly (*p* < 0.05) different from those for fruits linked to Filters 7 and 8, respectively, due to the effect of wetland contact and resting time variables. Mean iron concentrations in soil and fruits were significantly negatively correlated with each other (*R* = −0.475, *p* < 0.001). Moreover, regression analysis findings (Figure 6b) indicated that iron concentrations in the fruits decreased linearly but not significantly with the corresponding values in the soil due to their low bioavailability to the plants, as reported by FAO [23].

The iron CF in the tested fruits followed the order Filter 4 > Filter 6 > Filter 7 > Filter 2 > Filter 8 with percentage points of 1.8%, 1.5%, 0.8%, 0.6% and 0.3%, respectively. Compared with their mothers, chilli generation fruits had iron concentrations that were significantly (*p* < 0.05) lower (Supplementary Materials Table S4 and Figure 7b), highlighting the effect of higher irrigation volumes applied to the mother plants than those for the first generation plants [8]. Furthermore, iron levels in assessed fruits were below the standard of 425 mg/kg [48]. This metal is a vital element for human health, but excessive quantities may result in damage to tissues as reported by Abbaspour et al. [54].

#### 3.3.5. Potassium

Figure 5 shows that maximum mean potassium values were detected in fruits linked to Filter 8 effluent trailed by Filter 6 drain waters. In comparison, minimum concentrations were noted for fruits linked to Filter 2. However, potassium levels in fruits exceeded those numbers published previously [51]; 1870 mg per 100 g of sun-dried chillies. Fruits obtained from plants watered with Filter 2 (big gravel) outflow water had potassium values less than those of Filter 4 (small gravel). Fruits obtained from the latter filter had potassium concentrations that were similar to those of Filter 7 (Figure 5). Moreover, the Supplementary Materials Table S4 highlights that fruits obtained from chillies irrigated with effluent from Filters 4 and 7 had potassium values that were significantly (*p* < 0.05) lower than those of Filters 6 and 8 in this order.

The potassium CF in the tested fruits had this sequence: Filter 8 > Filter 6 > Filter 2 > Filter 7 > Filter 4 with points of 6826%, 5103%, 2768%, 2503% and 1897%, respectively. Compared with their mother plants (Figure 7), the first generation fruits showed some significant (*p* < 0.05) differences in mean potassium values (Supplementary Materials Table S4), possibly due to differences in element masses subjected to both plants [8] via irrigation water leading to differences in corresponding soil potassium concentrations available for uptake by pants. Potassium has an important role to play for plants, as it takes part in many physiological processes [23]. Moreover, this element is vital for human health, as it can maintain human blood pressure and water balance [48].

#### 3.3.6. Magnesium

Figure 5 illustrates that magnesium in the harvest was higher than 88 mg per 100 g of dried chillies as reported, previously [51]. The maximum magnesium values were detected in fruits obtained from chillies watered with Filter 8 effluent followed by Filter 6 effluent. In contrast, minimum concentrations were associated with Filter 2 effluent. Fruits picked from plants irrigated with Filter 2 effluent water had average magnesium values similar to those of plants associated with Filter 4, while the latter plants produced fruits of mean concentrations, which were less than those linked to Filter 7.

A statistical analysis (Supplementary Materials Table S4) indicated that there were significant (*p* < 0.05) dissimilarities in average magnesium values of chillies watered with Filter 7 outflow in comparison to those irrigated with effluent from Filter 8, indicating the importance of resting time on the wetland system. A significant (*p* < 0.05) dissimilarity was noted between fruit average values related to Filter 4 outflow compared to those corresponding ones linked to Filter 6. Moreover, regression analysis findings indicated that fruit magnesium concentrations linearly increased but not significantly with their corresponding levels in the soil (Figure 6c).

Furthermore, the magnesium CF in the tested fruits had this order: Filter 6 > Filter 8 > Filter 7 > Filter 2 > Filter 4 with corresponding percentages of 36%, 32%, 30%, 26% and 25%. Moreover, first chilli generation magnesium mean concentrations were observed to be different from their mother ones, as illustrated in Figure 1 and Supplementary Materials Table S4. However, the uptake of magnesium mainly depends on calcium and potassium as well as their levels in the soil. Plant magnesium uptake is usually a minor portion of the overall exchangeable magnesium available in the soil, which means that magnesium depletion from the soil by plant uptake is a minor factor, as discussed by Barber [52]. Magnesium is an essential plant nutrient as it is important for the photosynthesis process, because it is a building block of chlorophyll. The deficiency of magnesium in soil limits crop production [23]. Magnesium is an essential element for human health, as it is necessary for bone development. According to Musso [55], excessive magnesium in food may not cause any risk linked to the health of humans.

#### 3.3.7. Manganese

Manganese average values in the harvested plants are shown in Figure 5. Maximum levels were detected in fruits linked to Filter 8 effluent followed by Filter 6 drain water, while minimum concentrations were noted for those fruits linked to chillies watered with Filter 4 outflow. Supplementary Materials Table S4 indicates that fruit average manganese concentration values linked to plants irrigated with effluents from Filters 4 and 7 were significantly (*p* < 0.05) different from those irrigated with outflows associated with Filters 2 and 8, highlighting the impact of aggregate diameter and wetland resting time on corresponding soil element distribution available for uptake by plants [19]. Furthermore, fruits of plants irrigated with Filter 4 had average magnesium values, which were significantly (*p* < 0.05) dissimilar from those of Filter 6, indicating the effect of loading rate for the wetland system. However, differences in fruit manganese levels resulted from variations in this element within the corresponding soils (Figure 4). Correlation analysis results showed that manganese concentrations in the soil and fruits were negatively correlated with each other (*R* = −0.135, *p* = 0.206). Moreover, regression analysis results (Figure 6d) indicated that fruit manganese concentrations linearly decreased but not significantly with their corresponding values in the soil due to their low bioavailability as reported by Barber [52].

The manganese CF in the tested fruits had this sequence: Filter 6 > Filter 4 = Filter 2 = Filter 7 > Filter 8 with values of 17%, 13%, 13%, 13% and 12% in this order. In comparison to their mother chillies, the first generation chilli fruits showed mean manganese numbers that were significantly (*p* < 0.05) lower (Figure 7 and Supplementary Materials Table S4). Manganese is essential for chilli physiological processes, particularly photosynthesis. Manganese deficiencies lead to less yield, weak resistance against pathogens and lowered tolerance to droughts [56]. According to the threshold value of 500 mg/kg [48,57], findings indicate no exceedance in manganese of harvested fruits.

#### 3.3.8. Zinc

Figure 5 illustrates that maximum zinc values were noted in fruits obtained from chillies fed with Filter 4 effluent, while minimum values were recorded in chilli fruits watered with Filter 2 effluent. However, the observed zinc values were higher than 1.02 mg for 100 g of dried chillies, which was reported by Ciju [51]. The findings (Supplementary Materials Table S4) indicated that average zinc values in fruits linked to Filters 4 and 8 were significantly (*p* < 0.05) dissimilar from those for Filters 2 and 7, correspondingly, highlighting the effect of the gravel diameter and resting time on the associated soil zinc values, which subsequently resulted in considerable fruit zinc variation levels (Figure 4). No significant (*p* > 0.05) negative correlation relationship was found for zinc concentrations when comparing soils and fruits (Figure 6e).

The zinc CF order in the tested fruits was as follows: Filter 6 > Filter 4 > Filter 7 > Filter 8 > Filter 2 with values of 210%, 189%, 178%, 170% and 97%, respectively. However, first generation chilli plants had average zinc values, which were significantly (*p* < 0.05) unlike from their corresponding values obtained from their mothers (Figure 7 and Supplementary Materials Table S4). Zinc is an important micronutrient for chillies and controls gene expression [58]. Results showed that zinc levels in fruits obtained from all irrigated plants (with exception of those fed with Filter 2 effluent) exceeded the standard of 50 mg/kg [48,57]. The contamination/pollution (C P^−1^) ratio was evaluated using the tested fruit metal values divided by the corresponding standard values [59]. Based on listed intervals of C P^−1^ by Lăcătuşu [59], findings indicated that all tested chilli fruits for zinc were observed to be marginally polluted as C P^−1^ showed values from 1.1 to 2.0. According to FAO/WHO [48], zinc is important for human health. However, consuming high doses of zinc for a long time may cause toxicity effects.

#### 3.3.9. Boron

Figure 5 indicates that boron was detected at maximum levels in the fruits obtained from plants watered with effluent from Filter 6 (trailed by Filter 4). Minimum concentrations were determined for fruits from Filter 8-related plants. Chillies from Filters 2 and 7 had average boron concentrations that were lower than those of Filter 4, which was associated with boron concentrations lower than those for Filter 6. Moreover, chilli fruits irrigated with effluent from Filters 7 and 8 drain were different from each other (Figure 5). Results showed that boron concentrations in fruits and soils were negatively but not significantly correlated with each other as shown in Figure 6f, which indicates that boron in the fruits decreased linearly with their corresponding soil values.

The boron CF order in the tested fruits was like this: Filter 6 > Filter 7 > Filter 8 > Filter 2 > Filter 4 with values of 408%, 355%, 281%, 267% and 257%, correspondingly. These results contradict those reported by Diana [60] indicating that soil can contain various boron concentrations. However, only a small amount of boron can be obtained by plants. Therefore, it was not detected in the mother chillies. This is possibly due to the higher calcium concentrations in soils associated with the mother plants compared to those of the first generation plants, because of the higher irrigation water volume applied to the former [8], which will inhibit boron uptake and utilization [52]. Nevertheless, boron is a key micronutrient for chilli growth, as it has an important role in metabolism processes, mainly related to the stabilization of cell membranes [61,62]. Considering human health, boron positively affects the growth of bones and reducing the risk of getting cancer [63].

## 4. Conclusions

This work indicates that wetlands can be applied successfully for domestic sewage treatment. Moreover, the corresponding system effluent can subsequently be recycling for irrigating chillies used to treated wastewater, when grown in organic growth media. Total coliforms, ammonium-nitrogen, phosphorus and potassium significantly (*p* < 0.05) surpassed standards (1000 CFU/100 mL, 5 mg/L, 2 mg/L and 2 mg/L, respectively) set for irrigation. The highest corresponding values were 157,072 CFU/100 mL, 8.5 mg/L, 5.0 mg/L, and 7.0 mg/L in that order. No microbial content was observed in the harvested fruits. Reuse of treated wastewater for irrigation resulted in increases of some minerals like zinc, aluminum and magnesium in the soil compared to the raw materials. Recycling of purified wastewater for irrigation did not cause any significant soil mineral contamination. Slight contamination by zinc was noticed in the harvested chillies according to vegetable quality standards (threshold of 50 mg/kg). Zinc concentrations for Filters 2, 4, 6, 7 and 8 were 35.8, 60.6, 65.1, 65.5 and 53.2 mg/kg, respectively.

The element concentrations in the harvested fruits depended greatly on the corresponding concentrations and element bioavailability in the soil. Results indicated, for example, high concentration factor values for potassium (6826%) followed by zinc (210%), and a low value of 2% for iron.

The mother plants required a lot of irrigation water (pre-treated wastewater), because they were much taller (due to the plants not being adapted to high nitrogen loads in the irrigation water) and had higher biomass compared to the corresponding daughter plants, which also received the same type of irrigation water, but were apparently adapted to the high nitrogen load compared to their mother plants. This reduction in irrigation volumes concerning the first generation chillies (daughter plants) compared to their mothers led to a lowering in total element mass and consequently a reduction in first generation chilli fruit mineral content compared to their mother plants, increasing safety for human consumption.

Domestic wastewater treatment using reed beds and subsequent recycling of the treated wastewater for irrigation of crops such as chilli is a good substitute to the traditional use of drinking water in irrigation. This recycling approach helps to overcome the worldwide water scarcity problem by saving drinking water and reducing fertilizer application due to the various nutrients and minerals available in the treated wastewater.

The authors recommend long-term field studies on soil property changes as well as on plant growth, productivity and food safety using pre-treated wastewater from large-scale wetland systems. More replicates should be used for all plant generations.

## Figures and Tables

**Table 1:** Filter outflow (irrigation water for plants) water quality (mean ± standard deviation (number of samples)) compared to common standard values.

**Filter**	**COD**	**BOD_5_**	**DO**	**NH_4_-N**	**NO_3_-N**
Filter 2	45.0 ± 18.14 (19)	18.9 ± 14.98 (41)	2.8 ± 1.56 (45)	4.6 ± 4.57 (23)	1.6 ± 2.06 (22)
Filter 4	43.3 ± 13.10 (18)	15.6 ± 15.10 (42)	3.1 ± 1.96 (44)	2.9 ± 2.86 (22)	0.4 ± 0.48 (21)
Filter 6	63.9 ± 36.81 (18)	29.4 ± 26.99 (41)	2.9 ± 1.53 (45)	8.5 ± 7.30 (24)	3.9 ± 3.57 (24)
Filter 7	42.2 ± 23.82 (16)	14.2 ± 12.76 (48)	3.1 ± 2.13 (46)	3.5 ± 4.23 (17)	4.0 ± 3.36 (15)
Filter 8	60.4 ± 34.41 (17)	17.2 ± 13.46 (53)	3.1 ± 2.39 (50)	1.6 ± 2.33 (18)	3.0 ± 2.29 (16)
Standards *	-	-	-	5.0	30.0
**Filter**	**PO_4_-P**	**SS**	**NTU**	**pH**	**EC**
Filter 2	4.1 ± 1.47 (24)	12.1 ± 10.07 (53)	9.2 ± 8.07 (53)	6.6 ± 0.26 (52)	462.6 ± 146.89 (44)
Filter 4	3.7 ± 1.24 (23)	4.7 ± 6.25 (52)	3.5 ± 4.19 (51)	6.6 ± 0.28 (51)	483.4 ± 155 (43)
Filter 6	5.0 ± 2.01 (23)	11.0 ± 13.00 (53)	8.2 ± 9.05 (51)	6.8 ± 0.28 (52)	832.4 ± 298.17 (44)
Filter 7	4.3 ± 2.53 (19)	4.7 ± 8.16 (56)	3.8 ± 3.71 (55)	6.7 ± 0.32 (58)	582.9 ± 442.86 (44)
Filter 8	4.0 ± 2.09 (20)	3.1 ± 3.71 (56)	3.1 ± 2.76 (55)	6.6 ± 0.38 (61)	584.1 ± 459.11 (47)
Standards *	2.0			6.5-8.4	3000

Note: COD, chemical oxygen demand (mg/L); BOD_5_, biochemical oxygen demand (mg/L); DO, dissolved oxygen (mg/l); NH_4_-N, ammonia-nitrogen (mg/L); NO_3_-N, nitrate-nitrogen (mg/L); PO_4_-P, orthophosphate-phosphorus (mg/L); SS, suspended solids (mg/L); NTU, turbidity (NTU); EC, electrical conductivity (µS/cm); * Common standards such as FAO [1].

## Supplementary Materials

The following are available online at http://www.mdpi.com/1660-4601/15/8/1776/s1, Table S1: Statistically significant differences in properties of soil subjected to different irrigation water types, Table S2: Correlation coefficients and associated significances between soil elements using the non-parametric Spearman correlation, Table S3: Overview of the statistically significant differences for elements within the harvested fruits, Table S4: Differences in Chilli fruit mean element concentrations harvested from mother and generation plants.

## References

[1] FAO (2003). User’s Manual for Irrigation with Treated Wastewater.

[2] Pinto U., Maheshwari B.L., Grewal H.S. (2010). Effects of greywater irrigation on plant growth, water use and soil properties. Resour. Conserv. Recycl..

[3] Zavadil J. (2009). The effect of municipal wastewater irrigation on the yield and quality of vegetables and crops. Soil Water Res..

[4] Sani A., Scholz M., Bouillon L. (2013). Seasonal assessment of experimental vertical-flow constructed wetlands treating domestic wastewater. Bioresour. Technol..

[5] Scholz M. (2010). Wetland System—Storm Water Management Control.

[6] Almuktar S.A.A.A.N., Scholz M. (2016). Mineral and biological contamination of soil and *Capsicum annuum* irrigated with recycled domestic wastewater. Agric. Water Manag..

[7] Almuktar S.A.A.A.N., Scholz M. (2016). Experimental assessment of recycled diesel spill-contaminated domestic wastewater treated by reed beds for irrigation of Sweet Peppers. Int. J. Environ. Res. Public Health.

[8] Almuktar S.A.A.A.N., Abed S.N., Scholz M. (2017). Recycling of domestic wastewater treated by vertical-flow wetlands for irrigation of two consecutive *Capsicum annuum* generations. Ecol. Eng..

[9] Cirelli G.L., Consoli S., Licciardello F., Aiello R., Giuffrida F., Leonardi C. (2012). Treated municipal wastewater reuse in vegetable production. Agric. Water Manag..

[10] Aiello R., Cirelli G.L., Consoli S. (2007). Effects of reclaimed wastewater irrigation on soil and tomato fruits: A case study in Sicily (Italy). Agric. Water Manag..

[11] Chary N.S., Kamala C.T., Raj D.S.S. (2008). Assessing risk of heavy metals from consuming food grown on sewage irrigated soils and food chain transfer. Ecotoxicol. Environ. Saf..

[12] Almuktar S.A.A.A.N., Scholz M., Al-Isawi R.H.K., Sani A. (2015). Recycling of domestic wastewater treated by vertical-flow wetlands for irrigating Chillies and Sweet Peppers. Agric. Water Manag..

[13] Almuktar S.A.A.A.N., Scholz M., Al-Isawi R.H.K., Sani A. (2015). Recycling of domestic wastewater treated by vertical-flow wetlands for watering of vegetables. Water Pract. Technol..

[14] Almuktar S.A.A.A.N., Scholz M. (2015). Microbial contamination of *Capsicum annuum* irrigated with recycled domestic wastewater treated by vertical-flow wetlands. Ecol. Eng..

[15] FAO (1994). The State of Food and Agriculture.

[16] APHA (2005). Standard Methods for the Examination of Water and Wastewater.

[17] EPA (1994). Determination of Metals and Trace Elements in Water and Wastes by Inductively Coupled Plasma-Atomic Emission Spectrometry.

[18] Plank C.O. (1992). Plant Analysis Reference Procedures for the Southern Region of the United States.

[19] Bar-Tal A., Aloni B., Karni L., Rosenberg R. (2001). Nitrogen nutrition of greenhouse pepper. II. Effects of nitrogen concentration and NO3: NH4 ratio on growth, transpiration, and nutrient uptake. HortScience.

[20] Pescod M.B. (1992). Wastewater treatment and use in agriculture. FAO Irrigationand Drainage Paper Number 47.

[21] Scholz M. (2006). Wetlands Systems to Control Urban Runoff.

[22] McCauly A., Jones C., Jacobsen J. (2011). Plant Nutrient Functions and Deficiency and Toxicity Symptoms.

[23] FAO (1972). Trace Elements in Soils and Agriculture.

[24] Wiseman I.M., Edwards P.J. (2004). Constructed wetlands for minewater treatment: Performance and sustainability. Water Environ. J..

[25] Lesley B., Daniel H., Paul Y. (2008). Iron and manganese removal in wetland treatment systems: Rates, processes and implications for management. Sci. Total Environ..

[26] Rahimi Z., Aboutalebi A., Zakerin A. (2013). Comparison of different medium for production of sweet pepper transplant. Int. Res. J. Appl. Basic Sci..

[27] Millaleo R., Reyes-Díaz M., Ivanov A., Mora M., Alberdi M. (2010). Manganese as essential and toxic element for plants: Transport, accumulation and resistance mechanisms. J. Soil Sci. Plant Nutr..

[28] Yeh T.Y., Chou C.C., Pan C.T. (2009). Heavy metal removal within pilot-scale constructed wetlands receiving river water contaminated by confined swine operations. Desalination.

[29] Cakmak I. (2005). The role of potassium in alleviating detrimental effects of abiotic stresses in plants. J. Plant Nutr. Soil Sci..

[30] Deng H., Ye Z.H., Wong M.H. (2004). Accumulation of lead, zinc, copper and cadmium by 12 wetland plant species thriving in metal-contaminated sites in China. Environ. Pollut..

[31] Galletti A., Verlicchi P., Ranieri E. (2010). Removal and accumulation of Cu, Ni and Zn in horizontal subsurface flow constructed wetlands: Contribution of vegetation and filling medium. Sci. Total Environ..

[32] Guittonny-Philippe A., Masotti V., Höhener P., Boudenne J.L., Viglione J., Laffont-Schwob I. (2014). Constructed wetlands to reduce metal pollution from industrial catchments in aquatic Mediterranean ecosystems: A review to overcome obstacles and suggest potential solutions. Environ. Int..

[33] Travis M.J., Wiel-Shafran A., Weisbrod N., Adar E., Gross A. (2010). Greywater reuse for irrigation: Effect on soil properties. Sci. Total Environ..

[34] Kadlec R., Knight R. (1996). Treatment Wetlands.

[35] Green M., Friedler E., Ruskol Y., Safrai I. (1997). Investigation of alternative method for nitrification in constructed wetlands. Water Sci. Technol..

[36] Garcia J., Rousseau D.P., Morato J., Lesage E.L.S., Matamoros V., Bayona J.M. (2010). Contaminant removal processes in subsurface-flow constructed wetlands: A review. Crit. Rev. Environ. Sci. Technol..

[37] Hua G.F., Li L., Zhao Y.Q., Zhu W., Shen J.Q. (2013). An integrated model of substrate clogging in vertical flow constructed wetlands. J. Environ. Manag..

[38] Kadlec R.H., Wallace S.D. (2009). Treatment Wetlands.

[39] Sundaravadivel M., Vigneswaran S. (2001). Constructed wetlands for wastewater treatment. Crit. Rev. Environ. Sci. Technol..

[40] Manios T., Stentiford E.I., Millner P. (2003). Removal of total suspended solids from wastewater in constructed horizontal flow subsurface wetlands. J. Environ. Sci. Health Part A.

[41] Saeed T., Sun G. (2012). A review on nitrogen and organics removal mechanisms in subsurface flow constructed wetlands: Dependency on environmental parameters, operating conditions and supporting media. J. Environ. Manag..

[42] Maas E.V., Grattan S.R. (1999). Crop yields as affected by salinity. Agronomy.

[43] USEPA (2004). Guidelines for Water Reuse.

[44] Husson O. (2013). Redox potential (Eh) and pH as drivers of soil/plant/microorganism systems: A transdisciplinary overview pointing to integrative opportunities for agronomy. Plant Soil.

[45] Essington M.E. (2015). Soil and Water Chemistry: An Integrative Approach.

[46] EPA (1992). Guidelines for Water Reuse: Manual.

[47] Stahl T., Taschan H., Brunn H. (2011). Aluminium content of selected foods and food products. Environ. Sci. Eur..

[48] FAO/WHO (2001). Report on the 32nd Session of the Codex Committee on Food Additives and Contaminants.

[49] Li Q., Chen Y., Fu H., Cui Z., Shi L., Wang L., Liu Z. (2012). Health risk of heavy metals in food crops grown on reclaimed tidal flat soil in the Pearl River Estuary, China. J. Hazard. Mater..

[50] Kidd P.S., Proctor J. (2000). Effects of aluminum on the growth and mineral composition of *Betula pendula* Roth. J. Exp. Bot..

[51] Ciju R.J. (2013). Chile Peppers.

[52] Barber S.A. (1995). Soil Nutrient Bioavailability: A Mechanistic Approach.

[53] Zhu K., Prince R.L. (2012). Calcium and bone. Clin. Biochem..

[54] Abbaspour N., Hurrell R., Kelishadi R. (2014). Review on iron and its importance for human health. J. Res. Med. Sci..

[55] Musso C. (2009). Magnesium metabolism in health and disease. Int. Urol. Nephrol..

[56] Hakala M., Rantamäki S., Puputti E.M., Tyystjärvi T., Tyystjärvi E. (2006). Photoinhibition of manganese enzymes: insights into the mechanism of photosystem II photoinhibition. J. Exp. Bot..

[57] European Commission (2001). Setting Maximum Levels for Certain Contaminants in Food Stuffs.

[58] Brown P.H., Cakmak I., Zhang Q. (1993). Form and function of zinc plants. Zinc in Soils and Plants.

[59] Lăcătuşu R. (1998). Appraising levels of soil contamination and pollution with heavy metals. Land Information Systems.

[60] Diana G. (2006). Boron in the soil, from deficit to toxicity. Informatore Agrario.

[61] Cakmak I., Römheld V. (1997). Boron deficiency-induced impairments of cellular functions in plants. Plant Soil.

[62] Blevins D.G., Lukaszewski K.M. (1998). Boron in plant structure and function. Annu. Rev. Plant Biol..

[63] Nielsen F.H. (2014). Update on human health effects of boron. J. Trace Elem. Med. Biol..

