# Supplementary material for: Contaminations of Soil and Two Capsicum annuum Generations Irrigated by Reused Urban Wastewater Treated by Different Reed Beds
Source: Int J Environ Res Public Health. 2018 Aug 18;15(8):1776. doi: 10.3390/ijerph15081776 (PMC6121525; doi:10.3390/ijerph15081776)
Supplement: Supplementary file 1 [file ijerph-15-01776-s001.pdf]

Table S1. Statistically significant differences in properties of soil subjected to different irrigation water types.

**Shapiro-Wilk test (*p*-value)**[a]	**Statistical test**	***p*-values for treatment combinations**	**Treatment**	**Statistic (*p*-value)**[b]					
				Filter 2	Filter 4	Filter 6	Filter 7	Filter 8	Raw soil
Soil pH (-)									
0.180	ANOVA[d]	< 0.001	Filter 2	n.a.[c]	< 0.001	< 0.001	0.008	0.731	0.618
			Filter 4	< 0.001	n.a.c	< 0.001	< 0.001	< 0.001	0.025
			Filter 6	< 0.001	< 0.001	n.a.[c]	0.009	< 0.001	0.465
			Filter 7	0.008	< 0.001	0.009	n.a.[c]	0.070	1.000
			Filter 8	0.731	< 0.001	< 0.001	0.070	n.a.[c]	0.845
			Raw soil	0.618	0.025	0.465	1.000	0.845	n.a.[c]
Soil redox potential (mV)									
0.220	ANOVA[d]	< 0.001	Filter 2	n.a.[c]	< 0.001	< 0.001	0.007	0.735	0.931
			Filter 4	< 0.001	n.a.[c]	< 0.001	< 0.001	< 0.001	0.092
			Filter 6	< 0.001	< 0.001	n.a.[c]	0.008	< 0.001	0.250
			Filter 7	0.007	< 0.001	0.008	n.a.[c]	0.067	0.993
			Filter 8	0.735	< 0.001	< 0.001	0.067	n.a.[c]	0.995
			Raw soil	0.931	0.092	0.250	0.993	0.995	n.a.[c]

Table S1 (continued)

Soil electrical conductivity (µS /cm[f])									
< 0.001	Kruskal-Wallis	< 0.001	Filter 2	n.a.[c]	0.253	0.009	0.003	0.189	< 0.001
			Filter 4	0.253	n.a.[c]	< 0.001	< 0.001	0.014	< 0.001
			Filter 6	0.009	< 0.001	n.a.[c]	0.755	0.192	0.051
			Filter 7	0.003	< 0.001	0.755	n.a.[c]	0.106	0.100
			Filter 8	0.189	0.014	0.192	0.106	n.a.[c]	< 0.001
			Raw soil	< 0.001	< 0.001	0.051	0.100	< 0.001	n.a.[c]
Soil Total coliforms (CFU[g] /g)									
0.107	ANOVA[d]	< 0.001	Filter 2	n.a.[c]	< 0.001	< 0.001	0.343	0.998	nm
			Filter 4	< 0.001	n.a.[c]	0.027	0.005	< 0.001	nm
			Filter 6	< 0.001	0.027	n.a.[c]	< 0.001	< 0.001	nm
			Filter 7	0.343	0.005	< 0.001	n.a.[c]	0.214	nm
			Filter 8	0.998	< 0.001	< 0.001	0.214	n.a.[c]	nm
			Raw soil	–	–	–	–	–	n.a.[c]
Soil *Salmonella* spp. (CFU[g] /g)									
0.331	ANOVA[d]	< 0.001	Filter 2	n.a.[c]	< 0.001	< 0.001	0.071	0.972	nm
			Filter 4	< 0.001	n.a.[c]	0.956	0.015	< 0.001	nm
			Filter 6	< 0.001	0.956	n.a.[c]	0.003	< 0.001	nm
			Filter 7	0.071	0.015	0.003	n.a.[c]	0.020	nm
			Filter 8	0.972	< 0.001	< 0.001	0.020	n.a.[c]	nm
			Raw soil	–	–	–	–	–	n.a.[c]

Table S1 (continued)

Soil aluminium (mg/kg)									
0.015	Kruskal-Wallis[e]	< 0.001	Filter 2	n.a.[c]	0.539	0.846	0.130	0.106	0.003
			Filter 4	0.539	n.a.[c]	0.374	0.323	0.014	0.008
			Filter 6	0.846	0.374	n.a.[c]	0.061	0.118	0.001
			Filter 7	0.130	0.323	0.061	n.a.[c]	0.001	0.004
			Filter 8	0.106	0.014	0.118	0.001	n.a.[c]	< 0.001
			Raw soil	0.003	0.008	0.001	0.065	< 0.001	n.a.[c]
Soil calcium (mg/kg)									
0.028	Kruskal-Wallis[e]	< 0.001	Filter 2	n.a.[c]	0.023	0.013	0.579	0.009	0.163
			Filter 4	0.023	n.a.[c]	< 0.001	0.005	0.716	0.001
			Filter 6	0.013	< 0.001	n.a.[c]	0.053	< 0.001	0.465
			Filter 7	0.579	0.005	0.053	n.a.[c]	0.001	0.357
			Filter 8	0.009	0.716	< 0.001	0.001	n.a.[c]	< 0.001
			Raw soil	0.163	0.001	–	0.357	< 0.001	n.a.[c]
Soil iron (mg/kg)									
< 0.001	Kruskal-Wallis[e]	< 0.001	Filter 2	n.a.[c]	0.269	0.579	0.011	0.016	< 0.001
			Filter 4	0.269	n.a.[c]	0.539	0.110	< 0.001	< 0.001
			Filter 6	0.579	0.539	n.a.[c]	0.027	0.001	< 0.001
			Filter 7	0.011	0.110	0.027	n.a.[c]	< 0.001	< 0.001
			Filter 8	0.016	< 0.001	0.001	< 0.001	n.a.[c]	0.020
			Raw soil	< 0.001	< 0.001	< 0.001	< 0.001	0.020	n.a.[c]

Table S1 (continued)

Soil potassium (mg/kg)									
0.001	Kruskal-Wallis[e]	< 0.001	Filter 2	n.a.[c]	0.001	0.279	0.054	0.158	< 0.001
			Filter 4	0.001	n.a.[c]	0.032	0.023	0.070	0.020
			Filter 6	0.279	0.032	n.a.[c]	0.397	0.742	< 0.001
			Filter 7	0.054	0.023	0.397	n.a.[c]	0.605	0.006
			Filter 8	0.158	0.070	0.742	0.605	n.a.[c]	0.001
			Raw soil	< 0.001	0.020	< 0.001	0.006	0.001	n.a.[c]
Soil magnesium (mg/kg)									
0.040	Kruskal-Wallis[e]	< 0.001	Filter 2	n.a.[c]	0.203	< 0.001	0.430	0.037	< 0.001
			Filter 4	0.203	n.a.[c]	< 0.001	0.039	0.413	< 0.001
			Filter 6	< 0.001	< 0.001	n.a.[c]	0.005	< 0.001	0.021
			Filter 7	0.430	0.039	0.005	n.a.[c]	0.004	< 0.001
			Filter 8	0.037	0.413	< 0.001	0.004	n.a.[c]	< 0.001
			Raw soil	< 0.001	< 0.001	0.021	< 0.001	< 0.001	n.a.[c]
Soil manganese (mg/kg)									
0.001	Kruskal-Wallis[e]	< 0.001	Filter 2	n.a.[c]	0.222	0.058	0.178	< 0.001	< 0.001
			Filter 4	0.222	n.a.[c]	< 0.001	0.889	0.001	0.002
			Filter 6	0.058	< 0.001	n.a.[c]	< 0.001	< 0.001	< 0.001
			Filter 7	0.178	0.889	< 0.001	n.a.[c]	0.001	0.003
			Filter 8	< 0.001	0.001	< 0.001	0.001	n.a.[c]	0.681
			Raw soil	< 0.001	0.002	< 0.001	0.003	0.681	n.a.[c]

Table S1 (continued)

Soil zinc (mg/kg)									
0.090	ANOVA[d]	0.083	Filter 2	n.a.[c]	0.999	0.542	1.000	0.668	0.059
			Filter 4	0.999	n.a.[c]	0.847	0.999	0.908	0.192
			Filter 6	0.542	0.847	n.a.[c]	0.422	1.000	0.446
			Filter 7	1.000	0.999	0.422	n.a.[c]	0.595	0.120
			Filter 8	0.668	0.908	1.000	0.595	n.a.[c]	0.621
			Raw soil	0.059	0.192	0.446	0.120	0.621	n.a.[c]
Soil boron (mg/kg)									
0.001	Kruskal-Wallis[e]	<0.001	Filter 2	n.a.[c]	0.379	0.418	0.469	0.921	< 0.001
			Filter 4	0.379	n.a.[c]	0.091	0.109	0.327	0.006
			Filter 6	0.418	0.091	n.a.[c]	0.932	0.478	< 0.001
			Filter 7	0.469	0.109	0.932	n.a.[c]	0.532	< 0.001
			Filter 8	0.921	0.327	0.478	0.532	n.a.[c]	< 0.001
			Raw soil	< 0.001	0.006	< 0.001	< 0.001	< 0.001	n.a.[c]

[a] *p*-value : Test of normality (if *p*-value > 0.05, data are normally distributed; if *p*-value < 0.05, data are not normally distributed; [b] *p*-value: probability of the statistical test (values are statistically significantly different only if the *p*-value < 0.05 for the corresponding water quality parameter); [c] n.a: not applicable as the treatment compared with itself; [d] ANOVA: the parametric one-way analysis of variance test; [e] Kruskal-Wallis: the non-parametric Kruskal-Wallis test; mV: millivolts; [f] μS /cm: micro-Siemens per centimetre; [g] CFUg /g: colony forming units per gram; and nm: not measured.

Table S2. Correlation coefficients and associated significances between soil elements using the non-parametric Spearman correlation test.

**Element**	**Statistic**	**Element**							
		Aluminium	Calcium	Iron	Potassium	Magnesium	Manganese	Zinc	Boron
Aluminium	R[a]	1.000	0.686**	0.794**	0.811**	0.702**	0.768**	0.759**	-0.435
	p	n.a.[b]	0.000	0.000	0.000	0.000	0.000	0.000	0.092
Calcium	R[a]	0.686**	1.000	0.839**	0.894**	0.817**	0.915**	0.853**	0.021
	p	0.000	n.a.[b]	0.000	0.000	0.000	0.000	0.000	0.940
Iron	R[a]	0.794**	0.839**	1.000	0.909**	0.826**	0.890**	0.844**	-0.182
	p	0.000	0.000	n.a.[b]	0.000	0.000	0.000	0.000	0.499
Potassium	R[a]	0.811**	0.894**	0.909**	1.000	0.867**	0.871**	0.847**	-0.082
	p	0.000	0.000	0.000	n.a.[b]	0.000	0.000	0.000	0.762
Magnesium	R[a]	0.702**	0.817**	0.826**	0.867**	1.000	0.826**	0.821**	0.088
	p	0.000	0.000	0.000	0.000	n.a.[b]	0.000	0.000	0.745
Manganese	R[a]	0.768**	0.915**	0.890**	0.871**	0.826**	1.000	0.933**	-0.191
	p	0.000	0.000	0.000	0.000	0.000	n.a.[b]	0.000	0.478
Zinc	R[a]	0.759**	0.853**	0.844**	0.847**	0.821**	0.933**	1.000	-0.518*
	p	0.000	0.000	0.000	0.000	0.000	0.000	n.a.[b]	0.040
Boron	R[a]	-0.435	0.021	-0.182	-0.082	0.088	-0.191	-0.518*	1.000
	p	0.092	0.940	0.499	0.762	0.745	0.478	0.040	n.a

[a] R: correlation coefficient; *p*: probability of the statistical test (if *p*-value > 0.05, the variables are not statistically significantly correlated, if *p*-value < 0.05, the variables are statistically significantly correlated); [b] n.a: not applicable since the variable is tested to be correlated with itself (R = 1); **: correlation is significant at the 0.01 level; and *: correlation is significant at the 0.05 level.

Table S3 Overview of the statistically significant differences for elements within the harvested fruits.

**Shapiro-Wilk test (*p*-value)**[a]	**Statistical test**	***p*-values for treatment combinations**	**Treatment**	**Statistic (*p*-value)**[b]				
				Filter 2	Filter 4	Filter 6	Filter 7	Filter 8
Fruit calcium (mg/kg)								
0.008	Kruskal-Wallis[d]	< 0.001	Filter 2	n.a.[c]	0.936	< 0.001	< 0.001	< 0.001
			Filter 4	0.936	n.a.[c]	< 0.001	< 0.001	< 0.001
			Filter 6	< 0.001	< 0.001	n.a.[c]	0.034	0.006
			Filter 7	< 0.001	< 0.001	0.034	n.a.[c]	< 0.001
			Filter 8	< 0.001	< 0.001	0.006	< 0.001	n.a.[c]
Fruit iron (mg/kg)								
0.007	Kruskal-Wallis[d]	< 0.001	Filter 2	n.a.[c]	< 0.001	< 0.001	0.275	0.147
			Filter 4	< 0.001	n.a.[c]	0.852	< 0.001	< 0.001
			Filter 6	< 0.001	0.852	n.a.[c]	< 0.001	< 0.001
			Filter 7	0.275	< 0.001	< 0.001	n.a.[c]	0.012
			Filter 8	0.147	< 0.001	< 0.001	0.012	n.a.[c]
Fruit potassium (mg/kg)								
< 0.001	Kruskal-Wallis[d]	< 0.001	Filter 2	n.a.[c]	0.082	< 0.001	0.071	< 0.001
			Filter 4	0.082	n.a.[c]	0.030	0.948	0.009
			Filter 6	< 0.001	0.030	n.a.[c]	0.036	0.646
			Filter 7	0.071	0.948	0.036	n.a.[c]	0.011
			Filter 8	< 0.001	0.009	0.646	0.011	n.a.[c]

Table S3 (continued)

Fruit magnesium (mg/kg)								
0.124	ANOVA[e]	< 0.001	Filter 2	n.a.[c]	0.799	< 0.001	0.020	< 0.001
			Filter 4	0.799	n.a.[c]	< 0.001	0.280	< 0.001
			Filter 6	< 0.001	< 0.001	n.a.[c]	0.115	0.001
			Filter 7	0.020	0.280	0.115	n.a.[c]	< 0.001
			Filter 8	< 0.001	< 0.001	0.001	< 0.001	n.a.[c]
Fruit manganese (mg/kg)								
0.042	Kruskal-Wallis[d]	<0.001	Filter 2	n.a.[c]	0.009	0.019	0.881	0.010
			Filter 4	0.009	n.a.[c]	< 0.001	0.072	< 0.001
			Filter 6	0.019	< 0.001	n.a.[c]	0.028	0.826
			Filter 7	0.881	0.072	0.028	n.a.[c]	0.015
			Filter 8	0.010	< 0.001	0.826	0.015	n.a.[c]
Fruit zinc (mg/kg)								
0.012	Kruskal-Wallis[d]		Filter 2	n.a.[c]	< 0.001	< 0.001	< 0.001	0.001
			Filter 4	< 0.001	n.a.[c]	0.852	0.761	0.003
			Filter 6	< 0.001	0.852	n.a.[c]	0.914	0.007
			Filter 7	< 0.001	0.761	0.914	n.a.[c]	0.008
			Filter 8	0.001	0.003	0.007	0.008	n.a.[c]
Fruit boron (mg/kg)								
0.463	ANOVA[e]	<0.001	Filter 2	n.a.[c]	0.637	0.020	1.000	0.856
			Filter 4	0.637	n.a.[c]	0.136	0.417	0.045
			Filter 6	0.020	0.136	n.a.[c]	< 0.001	< 0.001
			Filter 7	1.000	0.417	< 0.001	n.a.[c]	0.469
			Filter 8	0.856	0.045	< 0.001	0.469	n.a.[c]

Table S3 (continued)

[a] *p*-value:

Test of normality (if *p*-value > 0.05, data are normally distributed; if *p*-value < 0.05, data are not normally distributed; [b] *p*-value: probability of the statistical test (values are statistically significantly different only if the *p*-value < 0.05 for the corresponding water quality parameter); [c] n.a: not applicable as the treatment compared with itself; [d] Kruskal-Wallis: the non-parametric Kruskal-Wallis test; and [e] ANOVA: the parametric one-way analysis of variance test.

Table S4. Differences in Chilli fruit mean element concentrations harvested from mother and generation plants.

**Element**	**Shapiro-Wilk test (*p*-value)**[a]	**Statistical test**	***P*-values between mother and generation plants**
Fruit calcium (mg/kg)			
Filter 2	< 0.001	M-W-U[c]	< 0.001
Filter 4	< 0.001	M-W-U[c]	< 0.001
Filter 6	< 0.001	M-W-U[c]	< 0.001
Filter 7	< 0.001	M-W-U[c]	< 0.001
Filter 8	0.001	M-W-U[c]	0.001
Fruit iron (mg/kg)			
Filter 2	< 0.001	M-W-U[c]	< 0.001
Filter 4	< 0.001	M-W-U[c]	< 0.001
Filter 6	< 0.001	M-W-U[c]	< 0.001
Filter 7	< 0.001	M-W-U[c]	< 0.001
Filter 8	< 0.001	M-W-U[c]	< 0.001
Fruit potassium (mg/kg)			
Filter 2	0.207	I-T[d]	0.229
Filter 4	0.814	I-T[d]	0.004
Filter 6	< 0.001	M-W-U[c]	0.261
Filter 7	0.004	M-W-U[c]	0.001
Filter 8	< 0.001	M-W-U[c]	0.261
Fruit magnesium (mg/kg)			
Filter 2	0.031	M-W-U[c]	1.000
Filter 4	0.283	I-T[d]	0.894
Filter 6	< 0.001	M-W-U[c]	< 0.001
Filter 7	0.179	I-T[d]	0.140
Filter 8	< 0.001	M-W-U[c]	< 0.001
Fruit manganese (mg/kg)			
Filter 2	< 0.001	M-W-U[c]	< 0.001
Filter 4	< 0.001	M-W-U[c]	< 0.001
Filter 6	< 0.001	M-W-U[c]	< 0.001
Filter 7	< 0.001	M-W-U[c]	< 0.001
Filter 8	< 0.001	M-W-U[c]	< 0.001
Fruit zinc (mg/kg)			
Filter 2	< 0.001	M-W-U[c]	< 0.001
Filter 4	0.001	M-W-U[c]	0.383
Filter 6	0.020	M-W-U[c]	0.018
Filter 7	0.012	M-W-U[c]	< 0.001
Filter 8	0.014	M-W-U[c]	< 0.001

Table S4 (continued)

[a] *p*-value: Test of normality (if *p*-value > 0.05, data are normally distributed; if *p*-value < 0.05, data are not normally distributed; [b] *p*-value: probability of the statistical test (values are statistically significantly different only if the *p*-value < 0.05 for the corresponding parameter); [c] M-W-U: the non-parametric Mann-Whitney U-test; and [d] I-T: the parametric Independent samples T-test.
